# Test-retest reliability of IPAQ environmental- module in an African population

**DOI:** 10.1186/1479-5868-5-38

**Published:** 2008-08-04

**Authors:** Adewale L Oyeyemi, Babatunde OA Adegoke, Adetoyeje Y Oyeyemi, Bukola M Fatudimu

**Affiliations:** 1 Department of Physiotherapy, College of Medicine, University of Ibadan, Nigeria; 2 College of Health Related Profession, State University of New York at Brooklyn, New York, USA

## Abstract

**Background:**

There is overwhelming evidence of the benefits of physical activity and the physical environment is increasingly recognized as a promising determinant of physical activity participation. The influence of the environment on physical activity has not been evaluated among black Africans and no specific measure exists for assessing environmental factors related to physical activity in an African environment. The IPAQ E- module was designed to assess environmental factors for physical activity participation and was considered to be relevant to all countries regardless of the stage of economic development. The objective of this study was to assess the test- retest reliability of IPAQ E- module in an African population.

**Methods:**

One hundred and three clinical students of a University in Nigeria were invited to participate in the reliability testing of IPAQ E- module. Sixteen of the 17- items on the environmental measure were assessed for test- retest reliability using intraclass correlation coefficient (ICC) with 95% Confidence interval (CI) overall and by gender. The measure addressed items regarding residential density, access to destinations, neighborhood infrastructures, aesthetic qualities, social environment, street connectivity and neighborhood safety.

**Results:**

Of the total respondents, 51.5% were males and 48.5% were females. Overall, the intraclass correlation coefficient (ICC) ranged from 0.43 to 0.91. The item regarding many interesting things to look at (aesthetic) produced the overall highest reliability score (ICC = 0.91, 95% CI = 0.86 – 0.94), while the item regarding safety from crime during the day (neighborhood safety) produced the lowest overall score (ICC = 0.43, 95% CI = 0.26 – 0.57). Reliability of items on neighborhood infrastructures ranged between substantial agreement to almost perfect agreement overall (ICC = 0.66 – 0.88) and by gender (male- ICC = 0.68 – 0.90 and female- ICC = 0.63 – 0.86). The access to destination items (ICC = 0.49 – 0.74), social environment (ICC = 0.62) and street connectivity (ICC = 0.78) all had acceptable reliability overall. Meaningful differences were found between males and females on two items on neighborhood safety and one item on access to destinations.

**Conclusion:**

The test- retest of IPAQ E- module resulted in moderate to almost perfect agreement for most of the items with few meaningful differences by gender. Environmental items of physical activity in an African population exhibited reliability similar to that in other environments. These results suggest that IPAQ E- module may be a useful measure for assessing environmental correlates of physical activity among population in Africa.

## Background

The overwhelming health benefits of physical activity are well documented [1,2]. There is however mounting evidence that physical activity in clearly defined context is on the decline worldwide [3] and the physical environment is increasingly being recognized as a potential and promising determinant of physical activity behaviour [4-7]. The influence of the physical environment on physical activity behaviour is currently unknown among the African population and no specific measure exists for the assessment of environmental correlates of physical activity in the African environment. However, the influence of the environment on physical activity behaviours is particularly important because physical activity occurs in specific environmental settings [8] and the environment that people build and inhabit provides potential opportunities for and barrier to engaging in a physically active lifestyle [9].

Until recently, studies on environmental correlates of physical activity have focused on the narrower interpersonal and individual levels of intervention while neglecting the broader contextual framework of socioecological model [10,11]. Research in this field are strengthened by utilizing the ecological model that recognizes the multiple levels of influence on health behaviours vis- social system, public policies and the physical environment [12]. This model has potential for explaining and facilitating better understanding of the influence of the environment on physical activity behaviors than the individual focus oriented model [12,13].

Sallis et al [14] highlighted the necessity of first identifying reliable and valid measures of theoretically relevant environmental variables before the influence of the environment on physical activity can be adequately evaluated. Most of the studies that have evaluated the psychometric properties of environmental measures were conducted among Caucasians, especially in the United States [14-17] and Europe [18,19], with their findings reflecting few differences in the reliability coefficients of the environmental variables. For instance, while the European studies [18,19] tend to identify items on neighborhood safety with lowest reliability coefficients, some American studies [16,17] implicated walking/cycling facilities and street/walking environments as items with the lowest reliability coefficients. There is therefore the potential possibility for the reliability of perceived environmental correlate items of physical activity to vary across countries and by cultures. Reliability studies from other continents may hence be necessary to fully compare and identify the international dimension and relevance of assessing environmental measures of physical activity behaviours.

Various measures for assessing environmental correlates of physical activity are in existence [14,17,18,20]. Although, the development of these measures were based on the contextual framework of socioecological model, they are mostly lengthy, voluminous and yet to be assessed internationally. The International Physical Activity Prevalence Study (IPS) group in 2002 developed a shorter survey (IPAQ Environmental- Module), primarily for the assessment of environmental factors for bicycling and walking in the neighborhoods. The strengths of IPAQ E- module are its brevity and the inclusion of variables that have been shown to be associated with different levels of physical activity in different countries. Also, items on the E- module were considered to reflect current thinking in the field of environmental correlates of physical activity that are considered to be relevant to all countries regardless of their stage of economic development [21]. This assertion may however, need to be tested in African countries where the physical environment is distinct from that in other parts of the world.

Evaluating the IPAQ E- module for reliability in an African environment is therefore necessary and may be a precursory step to identifying appropriate environmental correlates of physical activity behaviours among this population. Also, assessing the test- retest reliability of the IPAQ- E module in a cohort of African population may highlight differences, indicate cultural issues and espouse the environmental correlates that are contextually relevant to Africa. Since test- retest reliability is a useful means of assessing the reproducibility of a measure and hence the consistency and stability of an instrument over time [22], the purpose of this study was therefore to assess the test- retest reliability of the IPAQ E- module in an African population. The environment for the purpose of this study was defined as neighborhood characteristics.

## Methods

### Sample

Participants were undergraduate clinical students of the urban based premier University in Nigeria. They were selected from an ongoing larger study on environmental and sociodemographic determinants of physical activity among students of the University. A total of 298 male and female clinical students took part in the overall baseline survey consisting of 1006 students of the University. All the clinical students that were part of the baseline survey were invited to participate in the retest of the E-module questionnaire and about 69% of them (n = 103) agreed to participate in the retest study. Participants were given the second copy approximately 7 days after the first questionnaire was returned. The questionnaire was self administered and completed in the participants' rooms with the investigator in attendance in order to reduce items' misinterpretation. Socio-demographic information such as age, sex, height, weight, ethnic group, academic programs, years of study and religion were also sought from the participants. All participants provided an informed consent and the study was approved by the University of Ibadan/University College Hospital Joint Institutional Review Committee on Human Research (UI/EC/08/0004).

### Measurement of environmental characteristics

Sixteen self- report items from the IPAQ- Environmental Module (IPAQ E- module) designed for measuring environmental correlates of physical activity in the neighborhood were assessed for test- retest reliability in this study. The IPAQ E- module was made up of 17- environmental items that are grouped as core, recommended and optional. All core items were mandatory to be asked, while as many of the recommended items as possible should be asked in any study utilizing the E- module survey [21] [see Additional file 1]. One item from the recommended items "How many motor vehicles in working order are there in your household?" was not assessed due to the nature of the sample in the present study. Specifically, the study sample comprised students living in the University hostel and neighborhood was defined as the campus environment rather than their various household environments.

For the purpose of this study items on the IPAQ E- module were classified into seven categories [19]: residential density (one item), access to destinations (three items), neighborhood infrastructure (five items), aesthetic qualities (one item), social environment (one item), street connectivity (one item) and neighborhood safety (four items). These items have been shown to demonstrate moderate reliability coefficients among the Caucasians [17-19]. Responses to the IPAQ E- module were based on a 4- point likert scale ranging from strongly disagree to strongly agree as well as don't know or doesn't apply options for 15 of the questions. The only item with specific response option scale was the question assessing residential density (the main type of housing in my neighborhood).

### Data analysis

Analysis for the test-retest reliability of each of the environmental variables was conducted overall and by gender using the one- way model intraclass correlation coefficient (ICC) along with 95% confidence interval (CI). ICC represents the total variance in the measure (subject variability and measurement error) that was due to true differences between participants (subject variability) [15]. It accounts for the variability between, rather than within the participants. The agreement levels rating suggested by Landis and Koch: 0 – 0.2 (poor), 0.2 – 0.4 (fair), 0.4 – 0.6 (moderate), 0.6 – 0.8 (substantial) and 0.8 – 1.0 (almost perfect) was used to interpret the results [19]. Descriptive statistics of mean and percentage were used to describe the socio-demographic characteristics of the participants. Statistical analyses were performed with SPSS version 10.

## Results

One hundred and three participants completed the test- retest survey. The mean age and BMI of the participants were 24.24 ± 3.55 years and 23.24 ± 4.07 kg/m^2 ^respectively. About 51.5% were males and majority, (70.9%) was from the Yoruba ethnic group. More than half were clinical students of medicine (52.4%) and majority was of Christian religion (82.5%). The detailed general characteristics of the participants are shown in table 1.

**Table 1:** Characteristics of Participants

Characteristics	Male	Female	Total
	
	n (%)	n (%)	n (%)
**Gender**	53 (51.5)	50 (48.5)	

**Age (Years)**			
16 – 19	4 (3.9)	3 (2.9)	7 (6.8)
20 – 29	43 (41.7)	44 (42.7)	87 (84.5)
30 – 39	6 (5.8)	3 (2.9)	9 (8.7)

**BMI (kg/m^2^)**			
< 18.5	5 (4.9)	5 (4.9)	10 (9.7)
18.5 – 24.9	33 (32.0)	32 (31.1)	65 (63.1)
25.0 – 29.9	11 (10.7)	10 (9.7)	21 (20.4)
> 30.0	4 (3.9)	3 (2.9)	7 (6.8)

**Ethnic Group**			
Ibo	10 (9.7)	5 (4.9)	15 (14.6)
Hausa	0 (0)	3 (2.9)	3 (2.9)
Yoruba	33 (32.0)	40 (38.8)	73 (70.9)
Others	7 (6.8)	5 (4.9)	12 (11.7)

**Academic Programs**			
Physiotherapy	15 (14.6)	6 (5.8)	21 (20.4)
Medicine	27 (26.2)	27 (26.2)	54 (52.4)
Dentistry	11 (10.7)	17 (16.5)	28 (27.2)

**Years of Study**			
1^st ^Clinical	20 (19.4)	23 (22.3)	43 (41.7)
2^nd ^Clinical	19 (18.4)	15 (14.6)	34 (33.0)
3^rd ^Clinical	14 (13.6)	12 (11.7)	26 (25.2)

**Religion**			
Islam	9 (8.7)	9 (8.7)	18 (17.5)
Christianity	44 (42.7)	41 (39.8)	85 (82.5)

The result of the test- retest reliability for all respondents and by gender is presented in table 2 [see Additional file 2]. Overall, the one week ICC ranged from 0.43 – 0.91, with the lowest value recorded for question on crime during the day and the highest value for question on many interesting things to look at while walking. By gender, the ICC ranged from 0.11 – 0.96 for males and from 0.23 – 0.87 for females. Both males and females recorded the highest ICC on residential density item (main type of housing in the neighborhood), while two items on neighborhood safety that is, traffic against bicycling and traffic against walking demonstrated the lowest ICC among males and females respectively. When exploring the gender based findings, only items that differed by at least two categories on the rating of Landis and Koch were considered as meaningful and discussed as such.

### Reliability of items on residential density

The only question that assessed residential density was the main type of housing in the neighborhood. This item demonstrated almost perfect agreement overall (ICC = 0.89) and among the male participants (ICC = 0.96), but exhibited moderate agreement among the female participants (ICC = 0.56).

### Reliability of items on access to destination

The reliability of items on general access to destination ranged from moderate (ICC = 0.49) to substantial agreement (ICC = 0.74) overall. The lowest reliability was exhibited by the question "it is within 10- to- 15 minutes walk to the bus stop" and the highest reliability by the question "there are many shops within walking distance of university". The three items on access to destination differed meaningfully by gender. While males demonstrated poor agreement (ICC = 0.19) on the question "many shops are within walking distance of home", females tend to demonstrate almost perfect agreement (ICC = 0.80) on the question. Also, the question "many places to go within easy walking distance" was almost perfectly reliable (ICC = 0.89) among males but only moderately reliable (ICC = 0.59) among females. The question "it is within 10- to- 15 minutes walk to a bus stop from home" also demonstrated higher reliability (ICC = 0.60) among males than females (ICC = 0.27).

### Reliability of items on neighborhood infrastructure

Overall the five items pertaining to neighborhood infrastructures demonstrated substantial (ICC = 0.66) to almost perfect agreement (ICC = 0.88). The highest reliability was found for question on well maintained and unobstructed places for bicycling infrastructure. No meaningful gender differences were found in this domain except for the question on well maintained and unobstructed sidewalks in the neighborhood where higher reliability was found among males (ICC = 0.86) than among females (ICC = 0.56).

### Reliability of items on aesthetic qualities

The only question on aesthetic quality (many interesting things to look at while walking in the neighborhood) generated the highest reliability score overall (ICC = 0.91) and by female gender (ICC = 0.87). However, the reliability coefficients for both male (ICC = 0.94) and female (ICC = 0.87) fall within the same category (almost perfect).

### Reliability of items on social environment and street connectivity

For the social environment, seeing many people being physically active demonstrated substantial agreement (ICC = 0.62) overall with reliability somewhat higher among males (substantial agreement) than females (moderate agreement). Also, item on street connectivity (there are many four way intersections in the neighborhood) demonstrated substantial agreement (ICC = 0.78) overall with higher reliability among the male participants (almost perfect agreement) than their female counterparts (substantial agreement).

### Reliability of items on neighborhood safety

Apart from the question on crime rate at night (ICC = 0.83), all other items on neighborhood safety demonstrated moderate reliability overall, with the lowest reliability found for question on crime rate during the day (ICC = 0.43). Two items on neighborhood safety were meaningfully different by gender. Reliability was substantial (ICC = 0.69) among males but fair (ICC = 0.23) among females when assessing question on much traffic making it difficult or unpleasant to walk in the neighborhood. However, the reliability among females had moderate agreement (ICC = 0.45) while reliability among the males had poor agreement (ICC = 0.11) when assessing question on much traffic making it difficult or unpleasant to ride a bicycle.

## Discussion

This study evaluated the test- retest reliability of IPAQ E- module in an African population. The overall results indicate reliability to range from moderate agreement to almost perfect agreement. Few gender differences were observed in the reliability of some of the items on the E- module among the participants.

The highest reliability coefficients were found for items on aesthetic qualities and residential density such as "there are many interesting things to look at while walking in the neighborhood" and "the main type of housing in the neighborhood". Items on neighborhood safety such as "crime rate make it unsafe to go on walk during the day" and "so much traffic on the street makes it difficult or unpleasant to walk" demonstrated low reliability coefficient. This result reflects the stability of question pertaining to more objective features of the environment as aesthetic (trees, flowers, landscaping view etc) and types of housing (residential, office buildings, apartments etc) than subjective environmental features such as crime and safety that are easily overtaken by time and events. It is possible that participants in this study found it more difficult to subjectively assess crime rate and safety in their neighborhood, thereby reducing the reliability of the items. There is substantial interest in perceived crime as a correlate of physical activity behaviour and studies to date have produced inconsistent results on the association between perceived crime and physical activity [7,13,18,23]. Future studies may need to utilize more objective measures of crime and safety to identify the important relationship that exists between neighborhood safety and physical activity behaviours.

In a similar study [19] in Sweden, the main type of housing in the neighborhood was identified as the item with the highest reliability coefficient and neighborhood safety item "the crime rate makes it unsafe to go on walk during the day" demonstrated the lowest reliability coefficient. Also, in an American study [16], items on residential density were found to demonstrate high reliability while lower reliability was found for the question on unsafe walking during the day due to crime. Somewhat consistent with the higher reliability found for the item on aesthetic quality in this study, the question on many interesting things to look at while walking was found to have the highest reliability coefficient within the domain of items assessing neighborhood aesthetic [16]. The replication of these findings in an African population supports an international assumption that environmental variables pertaining to objective features like aesthetic qualities and housing type may be more reliable correlates of physical activity than subjective features like perceived crime and traffic.

In this study, environmental items on neighborhood infrastructures demonstrated good reliability coefficients that ranged from substantial agreement to almost perfect agreement. This finding may suggest stability of items assessing neighborhood infrastructures. This is likely because participants in this study lived in an environment where infrastructures like sidewalks and recreational facilities can readily be perceived as available but infrastructures like bicycle facilities may not be readily perceived as available. This can increase consistency and stabilize variation between responses thereby influencing good reproducibility of the items assessing neighborhood infrastructures. Similarly to the finding in the present study, Alexander et al [19] found substantial agreement in the reliability of all items on neighborhood infrastructures, while Brownson et al [16] found reliability of items assessing infrastructures for walking and cycling to vary from moderate agreement to substantial agreement. Somewhat consistently, another study from the United States [17] reported moderate agreement for walking and cycling facilities but found the subscale to demonstrate the lowest reliability when compared with other subscales. Also, in a Belgium study [18]; items on availability of sidewalks and bike lanes demonstrated almost perfect agreement. These findings suggest that variables assessing neighborhood infrastructures are ubiquitously reliable regardless of the geographical locations.

Several studies have implicated access to destination as an important correlate of physical activity [6,13,24]. In this study, the reliability of items on access to destination ranged from moderate agreement for the question "it is within 10- to -15 minutes walk to a transit/bus stop" to substantial agreement for the question "there are many places to go within easy walking distance". The lack of specificity in the time period given for the question "it is within 10 to 15 minutes walk to a transit stop" may reduce consistency between responses thereby lowering the reliability of the item. A previous study [18] that used a specific time and narrower definition of the neighborhood reported higher reliability coefficient for the same item. Also, the non attribution of time frame to the question "many places to go within walking distance" may explain its higher reliability when compared to other questions with time dimension. Consistently, a reliability study of similar sample size [20] reported comparable reliability coefficient for the question many places to go within walking distance (ICC = 0.63) to that of this study (ICC = 0.74). Similarly, the Sweden study [19] found reliability to range between (0.46 – 0.81) for items on access to destination, with particularly lower and higher reliability scores for the questions "it is within 10- to- 15 minutes walk to a transit stop from my house and "there are many places to go within easy walking distance" respectively. Also, items on land use mix- access (store within walking distance and easy walking to transit stop) and land use mix diversity (how long from home to get to business or convenience facilities) were found to demonstrate substantial to almost perfect reliability in an American study [17]. Replicating similar findings in an African population suggests an overwhelming importance of land use mix access as a reliable and viable environmental correlate of physical activity.

Substantial agreement was also found for the only item on social environment (seeing many people being physically active) and street connectivity (many four way intersections in the neighborhood). Similarly, the Sweden study [19] found substantial reliability for the only item on street connectivity but lower (moderate) reliability coefficient for the item pertaining to seeing many people being physically active in the neighborhood. However, an American study [16] found a somewhat lower reliability (ICC = 0.51) for the question on many four way intersections when compared to other studies. This was however not substantial since the value still falls within the moderate reliability found in these other studies [17,18].

Like the previous studies [19,20] that have assessed gender differences in the test- retest reliability of environmental measures of physical activity, few items demonstrated meaningful gender differences in the present study. Reliability was meaningfully higher among males than females on questions involving residential density, many places to go within easy walking distance, it is within 10- to- 15 minutes walk to the transit/bus stop and much traffic making it difficult or unpleasant to walk in the neighborhood. However, females had meaningfully higher reliability score on the questions "many shops within walking distance" and "so much traffic making it difficult or unpleasant to ride bicycle in the neighborhood" than males. Since males generally do not engage in shopping, it is plausible for them not to be consistent overtime in response to question on awareness of shops within walking distance of the university as compared to the females who may always be aware of shops because they regularly do shopping and hence answered more consistently, which consequently resulted in the meaningfully higher reliability of the item among females. While it may be difficult to draw any definite conclusion from these findings, it reflects the potential influence of gender on the reliability of some environmental correlate items. This is likely because evidence is now emerging that gender may exert a moderator link between perceived environment and physical activity behaviours [6,18,23].

### Limitations

This study has some important limitations. The generalization of this study is limited by the nature of the sample used which comprised clinical students of the University with potential higher comprehension and recall ability than may be found in the general population. The findings should therefore be interpreted with caution among other blacks of diverse educational level. Also, lower variation may exist in the limited environmental features available on campus than in the general community and this may lower reliability for the assessed environmental items in this study.

Like all other studies involving recall, it is possible for participants in the present study to give vague rather than factual responses to perceived environmental features in their neighborhood, thereby affecting results and inferences from this study. Also, though quite unlikely because of the short time frame, actual changes might have occurred between the retest surveys thereby reducing the observed reliability coefficients. The 95% confidence intervals for some of the reliability estimates, especially for the gender based findings were not precise thus suggesting the possibility of a low sample size.

The non utilization of objective environmental measures that would have served as the criterion for assessing the validity of IPAQ E- module in this study may constitute a limitation. Previous studies [18,25] have observed actual differences between objective and subjective data of environmental measures of physical activity. However, since no data presently exist in this field among the African population, the use of self- report measures of the environment may still hold sway for the time being in the African environment. This however does not suggest the non-importance of evaluating both subjective and objective environmental measures even at this early stage of this research type in African populations.

## Conclusion

This study has for the first time provided evidence on the psychometric properties of items on an environmental measure of physical activity in an African population. The test- retest reliability of IPAQ E- module ranged from moderate agreement to almost perfect agreement with few meaningful differences by gender. The reliability coefficients of environmental items among this population were mostly similar to that in other environments. Items on IPAQ E- module is therefore promising and may be useful for assessing environmental correlates of physical activity among the black population in Africa. Future studies should consider testing IPAQ E-module along with more objective environmental measures in a diverse African population.

## Authors' contributions

ALO conceived and designed the study, analyzed and interpreted the data, drafted the manuscript and gave approval for the final version. BOA was involved in study design, interpretation of data and drafting of the manuscript. AYO was involved in study design and drafting of the manuscript. BMF was involved in data acquisition and read the manuscript for final approval. All authors read and approved the final manuscript.
